# The Neural Correlates of Cued Reward Omission

**DOI:** 10.3389/fnhum.2021.615313

**Published:** 2021-02-11

**Authors:** Jessica A. Mollick, Luke J. Chang, Anjali Krishnan, Thomas E. Hazy, Kai A. Krueger, Guido K. W. Frank, Tor D. Wager, Randall C. O’Reilly

**Affiliations:** ^1^Department of Psychiatry, Yale University, New Haven, CT, United States; ^2^Department of Psychological and Brain Sciences, Dartmouth College, Hanover, NH, United States; ^3^Department of Psychology, Brooklyn College, City University of New York, Brooklyn, NY, United States; ^4^eCortex, Inc, Boulder, CO, United States; ^5^UCSD Eating Disorder Center for Treatment and Research, University of California, San Diego, San Diego, CA, United States; ^6^Department of Psychology and Computer Science Center for Neuroscience, University of California, Davis, Davis, CA, United States

**Keywords:** conditioned inhibition, habenula, fMRI, negative, prediction error, reward, learning

## Abstract

Compared to our understanding of positive prediction error signals occurring due to unexpected reward outcomes, less is known about the neural circuitry in humans that drives negative prediction errors during omission of expected rewards. While classical learning theories such as Rescorla–Wagner or temporal difference learning suggest that both types of prediction errors result from a simple subtraction, there has been recent evidence suggesting that different brain regions provide input to dopamine neurons which contributes to specific components of this prediction error computation. Here, we focus on the brain regions responding to negative prediction error signals, which has been well-established in animal studies to involve a distinct pathway through the lateral habenula. We examine the activity of this pathway in humans, using a conditioned inhibition paradigm with high-resolution functional MRI. First, participants learned to associate a sensory stimulus with reward delivery. Then, reward delivery was omitted whenever this stimulus was presented simultaneously with a different sensory stimulus, the conditioned inhibitor (CI). Both reward presentation and the reward-predictive cue activated midbrain dopamine regions, insula and orbitofrontal cortex. While we found significant activity at an uncorrected threshold for the CI in the habenula, consistent with our predictions, it did not survive correction for multiple comparisons and awaits further replication. Additionally, the pallidum and putamen regions of the basal ganglia showed modulations of activity for the inhibitor that did not survive the corrected threshold.

## Introduction

While the field of reinforcement learning has generally focused on the role of reward prediction errors in training reward expectations, the mechanisms involved in learning about omission of expected reward delivery are less well understood. Classical models of learning such as Rescorla–Wagner and TD models suggest that prediction errors result from a simple subtractive computation, which also has been shown to match the firing of dopamine neurons. However, there is also recent evidence suggesting that brain areas projecting to dopamine neurons may provide input which contributes to specific parts of this computation, for example, some regions may encode the level of expected reward (Cohen et al., 2012), while others may respond specifically to worse than expected outcomes. Here, we focus on the latter computation, which has been well-established in animal studies, showing that neurons in the lateral habenula respond both to aversive outcomes and the omission of an expected reward, and further drive an inhibition of dopamine neurons, leading to the “dip” component of prediction error encoding how much worse something was than expected (Matsumoto and Hikosaka, 2009b).

In appetitive Pavlovian conditioning, individuals learn expectations about stimuli that are reliably paired with rewards. This conditioning procedure causes the previously neutral cue to drive a conditioned response. In conditioned inhibition, a conditioned stimulus (CS) associated with reward is presented simultaneously with a conditioned inhibitor (CI), which causes the expected reward not to occur. Conditioned inhibition occurs because the unexpected omission of reward causes a negative reward prediction error. By learning theories like Rescorla–Wagner, if another sensory stimulus is reliably present during these unexpected omissions, the accumulation of negative prediction errors causes the CI to acquire negative value. This results in inhibitory conditioning, and a reduction of the conditioned response. For example, imagine that you enjoy drinking tea, but cannot make it when your kettle is broken. Over time, the broken kettle becomes a CI because it reliably predicts the omission of tea.

Computationally, conditioned inhibition is an interesting problem, because it relies on the negative prediction errors that occur when the CS+ is unexpectedly followed by a reward omission in the presence of the inhibitor, which causes the CI to acquire negative value, even though the CI has never been paired with an aversive stimulus. Once inhibition is acquired, the inhibitor can pass the summation test, meaning there is a reduced conditioned response to a CS paired with the inhibitor compared to the CS alone (Rescorla, 1969a). Further, we chose the paradigm based on the potential to dissociate the mechanisms of reward prediction at the time of the CS from those controlling reward predictions at the time of the unconditioned stimulus (US). In the trials where the inhibitor is presenting concurrently with the CS+, there may be a representation of the CS+ linked with an expectation of reward, along with a representation of the inhibitor linked with a reward omission. Interestingly, Tobler et al. (2003) showed a combined burst and dip to the CS+ paired with the Inhibitor, which may reflect these two associations. In contrast, at the time of the US, the conditioned inhibition procedure leads to an expectation of no reward, evidenced by the ability of the CI to transfer inhibition to a novel CS+, and the enhanced dopamine burst when the CI is unexpectedly followed by reward (Tobler et al., 2003). This account was recently simulated in a computational model of conditioned inhibition and other conditioning phenomena incorporating separate learning mechanisms for the control of dopamine responses at the time of the CS and US (Mollick et al., 2020). However, this theoretical account does not incorporate the idea that there might be learning for the combined stimulus of CS+ and Inhibitor as well, signaling a new context of reward omissions, drawing on ideas of state-splitting that may also occur in extinction (Redish et al., 2007), or as a conjunctive representation, possibly represented in the hippocampus (Rudy and O’Reilly, 2001).

The prediction error response in dopamine neurons includes both increases in firing for better than expected outcomes and decreases in firing, or dopamine dips, for worse than expected outcomes. However, few studies have focused on understanding the role of certain brain areas in the processes driving dopamine dip signals for worse than expected outcomes and how these areas are involved in learning about stimuli that predict reward omissions. In particular, an unanswered question remains about the extent to which brain areas involved in learning about reward omissions overlap with those involved in learning about aversive stimuli.

Theories about how the positive and negative valence learning systems interact have proposed that something that stops a negative state leads to positive emotions, while the omission of a positive reward leads to negative emotions (Mowrer, 1956; Solomon and Corbit, 1974; Seymour et al., 2007b; Maia, 2010). However, human fMRI studies have generally focused on the neural correlates of positive prediction errors for reward outcomes, though some have also begun to examine whether regions like the lateral habenula (Hennigan et al., 2015) and periaqueductal gray (PAG) (Roy et al., 2014) encode prediction error signals for aversive outcomes. While these studies have greatly advanced our understanding of the brain areas involved in learning about reward and aversion, they do not examine whether the same brain areas that are involved in associations of conditioned stimuli with rewards are the same regions that drive prediction errors if reward expectations are violated. Further, the diffuse modulatory effects of dopamine release make it difficult to tell whether the brain areas that encode reward prediction errors are providing inputs to the dopamine system or reflecting downstream effects of dopamine release. We ran a conditioned inhibition paradigm to look specifically at the negative prediction error mechanisms associated with learning about a predictor of reward omissions and compare those with learning signals for positive reward predictors.

While previous fMRI studies have looked at the neural mechanisms involved in monetary losses and the presentation of aversive stimuli, no human fMRI studies have focused on the learning about predictors of reward omissions in a conditioned inhibition experiment. Dopamine neurons respond to a CI with an inhibition, or pause in tonic firing, the same pattern of dopamine release in the substantia nigra seen to an aversive stimulus (Tobler et al., 2003; Schultz, 2007). Intriguingly, recent research has shown that this inhibition of dopamine neurons, or dip, is driven by the lateral habenula, which has been found to be activated during aversive processing and reward omissions (Matsumoto and Hikosaka, 2009b). In this study, we examined if the same signals that have been reported for reward omissions in monkey studies, particularly an increase in lateral habenula activity accompanied by a reduction in the firing of dopamine neurons, could be observed in human fMRI. These signals also occur for a CS associated with reward omission, so we predicted a strong habenula signal for the CI that was associated with reward omission.

To examine the brain areas involved in each of these computations, we ran a novel fMRI study, adapting the conditioned inhibition paradigm from Tobler et al. (2003) to human participants. Using a taste pump apparatus, participants learned to associate previously neutral visual stimuli with the presentation of orange juice rewards. In an initial conditioning block, participants learned associations of a CS+ with the orange juice reward and a CS− with the neutral solution. Importantly, this was then followed by a conditioned inhibition procedure, where the originally rewarded stimulus was paired with another cue that deterministically leads to the neutral solution instead of the expected orange juice reward. Due to the disappointment (and negative reward prediction errors) resulting from omission of the expected orange juice, the cue that predicts omission becomes a CI. Importantly, once a cue has acquired inhibitory properties, it should both reduce the value of the CS+ during the predictive phase, and lead to an expectation of no reward at the time of the US. Further, these inhibitory properties can be tested by unexpectedly following the CI with a juice reward. If it has acquired inhibition, then the unexpected presentation of reward after the inhibitor should lead to a prediction error signal. Further, the prediction error for the inhibitor followed by an unexpected reward should be larger than the prediction error that occurs when a neutral control stimulus is unexpectedly followed by reward, due to the inhibitory properties acquired by the inhibitor during conditioned inhibition.

**FIGURE 1:**
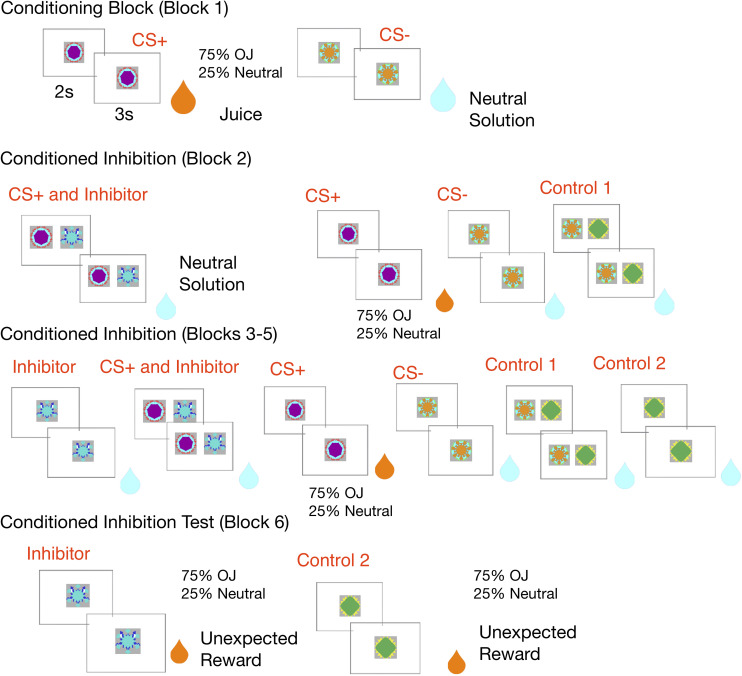
Experimental design: In the conditioning block, the CS+ is paired with an orange juice reward 75% of the time and a neutral solution 25% of the time, while a control CS– is always paired with neutral solution. In the conditioned inhibition block, the CS+ is paired with an inhibitor which leads to reward omission. The rewarded CS+ continues to be shown. This is followed by subsequent conditioned inhibition blocks, where the inhibitor is shown alone in a subset of trials, along with neutral controls. The experiment ends with a conditioned inhibition test where the inhibitor is unexpectedly followed by a juice reward, and the second control stimulus is also unexpectedly followed by reward.

See Figure 1 for a schematic of the conditioned inhibition fMRI design.

## Materials and Methods

### Participants

Nineteen participants (13 female) ranging between 19 and 55 years old, from the University of Colorado, Boulder, and the local community volunteered for the study. All participants were right-handed and generally in good health. Participants were screened for MRI contraindications and provided informed written consent for protocols approved by the Institutional Review Board of the University of Colorado, Boulder. Participants were paid $48 for completing the study in addition to earnings from the task.

### Experimental Procedures

**FIGURE 2:**
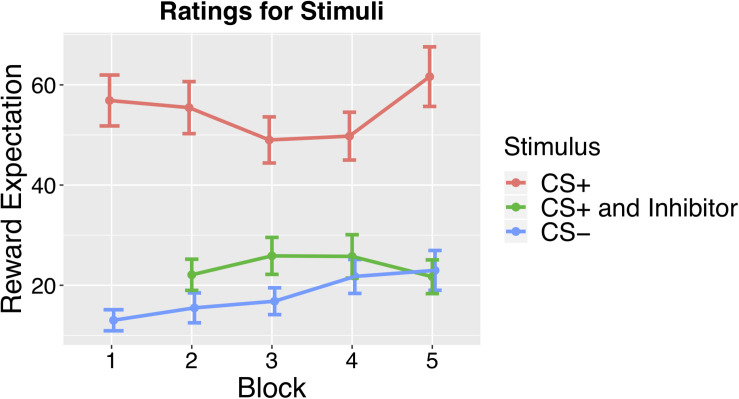
Results from the Monetary Reward Task conducted as a follow-up study. Mean ratings across subjects for CS+ CS–, and CS paired with the Inhibitor across blocks, which revealed significantly lower ratings for the CS+ paired with the Inhibitor than the CS+ alone.

The functional imaging was divided into six scanning runs, with an average length of 9 min, with brief 1–2 min breaks between blocks. The first 10 volumes of each run were discarded to account for equilibration of the scanner’s magnetic field. The experimental design is shown visually in Figure 2, and Supplementary Table S8 provides a schematic overview of trial types in each block.

In the first conditioning block of 48 trials, which lasted 8.4 min, participants were exposed to the initial CS – US contingencies, with equal number of CS+ and CS− trials. During Pavlovian conditioning, one fractal stimulus (CS+) was associated with reward (orange juice) 75% of the time, while another fractal (CS–) was deterministically associated with a neutral outcome (artificial saliva, 0.0116 g KCl, 0.0105 g NaHCO3 per 500 ML/water) (O’Doherty et al., 2003; Frank et al., 2012). This first conditioning block lasted 8.4 min.

This was followed by four conditioned inhibition blocks (blocks 2 -5), which consisted of 200 trials total, where the CS+ was paired with another stimulus, the CI, in 22% of these trials (44 trials). Presentation of the CI was deterministically associated with the presentation of the neutral solution, negating the reward prediction elicited by the CS+. In another 22% of total trials, participants continued to experience the initial CS – US pairing, with reward presented in 75% of these trials in order to keep the reward association from being extinguished by the conditioned inhibition procedure. The remaining trials consistent of several different neutral control stimuli (50% of total trials), and the CI viewed alone (6% of total trials).

This conditioned inhibition training was broken into several different blocks, based on the same sequence of trials as the original Tobler et al. (2003) paper. The first block of conditioned inhibition lasted 5.7 min and consisted of 32 trials, 8 each of CS+, CS–, and the CS+ paired with the Inhibitor, and an additional CS– control (consisting of two fractal images).

In blocks 3–5, which each lasted 10.4 min, and consisted of 168 total trials, participants saw five different stimuli, the CS+, CS–, CS+ paired with the Inhibitor, the CS– control consisting of two fractal images, the Inhibitor, and another CS– control consisting of a single fractal image. Each of these blocks consisted of 56 trials, 12 trials each of the CS+, CS–, CS+ paired with the Inhibitor, and the CS– control consisting of two images. Each block also included four trials where the Inhibitor was viewed alone and four trials of the CS– control consisting of a single fractal image.

The inhibitor was shown less frequently alone (1:3 ratio compared to other trials) to minimize learning about the inhibitor in isolation, which would have reduced the strength of the inhibitory procedure, as done in a previous conditioned inhibition study in monkeys (Tobler et al., 2003) which also included a block of conditioned inhibition before the inhibitor was viewed alone. The order and type of trials in each block was based off of the design in this original study.

In the final block, we ran an inhibition test block, which lasted 8.4 min and consisted of 48 trials. In the test block, we followed the CI with an unexpected juice reward 75% of the time, in order to test for positive reward prediction errors. A control CS– was also paired with an unexpected juice reward 75% of the time in the conditioned inhibition test block, and we expected less of a prediction error signal for the CS– paired with reward than the inhibitor since it did not develop an association with reward omission.

In each trial, there was a presentation of a fractal CS for 2 s. This was followed by 2 mL of orange juice (Tropicana brand) or the neutral-tasting solution, which was delivered by a taste pump connected to the stimulus computer. The onset of taste delivery was logged, and delivery of solution after receiving the trigger took about 3 s. It was a delay conditioning paradigm, where the CS remained onscreen until the US delivered was completed. After each trial of CS and US presentation, there was an ITI randomly sampled to be between 4 and 8 s. We selected participants who reported a preference for orange juice in the prescreening interview. Further, presentation of orange juice has been associated with higher pleasantness ratings than artificial saliva in prior studies (Takemura et al., 2011). Visual stimuli were presented with a projector inside the fMRI head coil.

There were several features of the design that were motivated by careful consideration of the learning problem. For example, we wanted to keep the duration between the CS and juice reward (US) consistent because there is evidence the striatum responds to temporal prediction errors (McClure et al., 2003). In addition, we chose a delay conditioning paradigm, where the CS remains onscreen while the US is delivered, because there has been considerable evidence that trace conditioning, which involves showing a CS that is removed before reward is delivered, depends on the integrity of the prefrontal cortex and hippocampus (Kronforst-Collins and Disterhoft, 1998), and we wanted to focus on the role of subcortical regions in conditioning. In addition, it is worth noting that the final block of conditioned inhibition, which we call an “inhibition test,” was designed to replicate a specific condition in the Tobler et al. (2003) study which compared the prediction errors for the CI followed by an unexpected reward with the prediction errors for a control stimulus. However, notably, behavioral tests of conditioned inhibition have suggested that two additional tests are important for assessing conditioned inhibition, an inhibitor should suppress responding to a CS+ when presented together (summation test), and also acquire conditioned excitatory properties more slowly when paired with a US in a retardation test (Rescorla, 1969b; Sosa and Ramírez, 2019).

### Data Acquisition

Magnetic-resonance imaging (MRI) data were acquired at the Center for Innovation and Creativity at CU Boulder using a 3T Siemens Trio scanner and a 32-channel receive-only head coil. To guide the functional imaging, a structural volume of the entire brain was acquired first using a T1-weighted magnetization-prepared rapid gradient-echo (MPRAGE) sequence [repetition time (TR): 2530 ms, echo time (TE1: 1.64 ms, TE2: 3,5 ms), flip angle (FA): 7°, voxel: 1 × 1 × 1-mm isotropic, field of view (FOV): 2.29 × 2.29 × 2 mm].

High-resolution functional images were acquired with a blood-oxygen-level-dependent (BOLD) contrast using a T2^∗^-weighted gradient-echo echo-planar imaging (EPI) sequence [TR: 1300 ms, TE: 25 ms, 75%, acceleration factor: 2, 22 cm FOV, in-plane voxel size: 2.29 mm, slice thickness 2 mm, no gap (voxel-size: 2.29 × 2.29 × 2 mm)]. With these parameters, 24 contiguous slices were collected in interleaved-ascending order for each volume. Slices were aligned parallel to the base of the OFC. Due to the focus of our study on subcortical areas, we acquired limited coverage, which included the amygdala, insula, midbrain, thalamus, striatum, and ventral prefrontal cortex.

The functional imaging was divided into six scanning runs, with an average length of 9 min. The first 10 volumes of each run were discarded to account for equilibration of the scanner’s magnetic field.

### Monetary Reward Task: Follow Up Study

In order to investigate whether there were behavioral effects of conditioned inhibition, we ran a follow-up study to look at how the conditioned inhibition procedure affected reward expectation. The study had an identical design, but used monetary rewards, and allowed us to look at the behavioral effects of conditioned inhibition by having participants rate their expectation of reward at the end of each training block. As our behavioral measure of conditioning, we assessed the reward expectation for each stimulus at the end of each block using a continuous rating scale for reward expectation, ranging from No Expectation to Strongest Expectation. Subject saw each CS in succession, followed by the rating scale depicted in Supplementary Figure S5, which was adapted from rating scales that have previously been validated for affective ratings across many modalities (Bartoshuk et al., 2004).

### Preprocessing

The preprocessing pipeline followed a well-validated preprocessing pipeline that has been used in several other studies (Wager et al., 2013; Woo et al., 2014), and is available online^1^, but with a distinct warping step. The first 10 images were discarded to account for the stabilization of the BOLD signal. Then, the functional images were motion-corrected using the realignment procedure in SPM 8, using a rigid-body, affine (six parameter) registration that helps correct for head movement during scanning. To identify outliers, we computed the mean and standard deviation across voxels for each image for all slices, and then calculated the Mahalanobis distance of each mean and standard deviation value, considering any volumes with a significant χ^2^ value as outliers, per the procedure described in Wager et al. (2013).

Next, these motion-corrected functional images were co-registered to the structural images using FSL’s epi_reg script, an affine co-registration that improves registration by segmenting the structural and functional images (Jenkinson and Smith, 2001; Jenkinson et al., 2002). Each structural T1 image was warped to standard space using the Advanced Normalization Toolbox (ANTs) (Avants et al., 2014). We then combined the transformation matrix from the functional to structural transformation with the warping matrix from the transformation of the structural to standard space to warp the functional data into standard space. After the transformation, a 4 mm FWHM Gaussian smoothing kernel was applied to the images.

The functional images were corrected for slice timing to account for acquiring slices at slightly different timepoints and then motion corrected using the realignment procedure in SPM8. Each outlier image detected by the Mahalanobis distance method was modeled as a nuisance covariate, by inserting a dummy code variable of 1 where the spike occurred.

In addition, we calculated several regressors of non-interest, which included an intercept for each run, dummy regressors for outlier images calculated by the spike detection method above, and motion-related covariates, which included 6 mean-centered motion parameter estimates, their squared values, successive differences and squared successive differences. Additional nuisance regressors were calculated by determining the first five principal components from the signal in the ventricles in the warped functional images with a 4 mm smoothing kernel.

### fMRI Analysis

The fMRI analysis involved separate regressors for each of the different stimuli in the experiment, including separate regressors for each of the stimuli in the conditioning, conditioned inhibition and inhibition test phases, to allow us to assess the effects of Pavlovian conditioning and conditioned inhibition on brain activity. To this end, we generated separate first-level model task regressors for the CS+ and the CS– in the first conditioning block. In the three following conditioned inhibition blocks, we created first-level model task regressors for the CS+, CS+ paired with the Inhibitor, and Inhibitor viewed alone, as well as the two other CS– stimuli. In the conditioned inhibition test blocks, we generated separate regressors for the Inhibitor and the CS–.

In the same first-level model, we also modeled each of the different outcomes following the CS with separate regressors, to allow us to examine the effects of expectation on outcome activity. Therefore, we generated separate regressors for the expected presentation of reward following the CS+, the unexpected reward omission resulting from presentation of the neutral solution following the CS+ (omission), and presentation of the neutral solution following each CS– (as a control stimulus for the effects of taste stimulation). In each case, the duration of each CS event was set to 2 s, while the duration of each US event was set to 3 s. The fixation cross, which was presented between each CS-US trial, was explicitly not modeled and considered the implicit baseline.

Further, to assess the effectiveness of the conditioned inhibition procedure, we ran a conditioned inhibition test where the inhibitor and the CS– control were unexpectedly paired with reward in the last block of the experiment. Therefore, the first level model also included separate regressors for both cue and outcome activity in this inhibition test block. If conditioned inhibition was successful and caused the inhibitor to acquire negative value, positive prediction errors should result when it is unexpectedly followed by a reward.

To specifically examine this, we looked separately at outcome activity when the Inhibitor was unexpectedly paired with reward and the trials where the Inhibitor was paired with no reward. Similarly, we also modeled the trials in the inhibition test block where the CS– was unexpectedly paired with reward separately from the trials where the CS– was followed by the expected neutral solution (no reward).

For the group level GLM analysis, we used a robust regression procedure, which has been shown to decrease sensitivity to outliers (Wager et al., 2005). Whole brain results were corrected for multiple comparisons with *q* < 0.05, FDR (false-discovery rate).

### ROI Analysis

We defined ROIs according to probabilistic atlases, whenever possible. For each anatomical atlas, we used a threshold to include only voxels that had 75% or higher probability. For the habenula ROI, we used the habenula ROI from a high-resolution atlas of the thalamus based on histological data (Krauth et al., 2010). Recent papers on defining the habenula in human fMRI suggest that total habenula volume (medial and lateral) is around 31–33 mm, approximately the size of a single voxel in standard fMRI protocols (Lawson et al., 2013). For this reason, we cannot differentiate between medial and lateral habenula in the ROI analysis. For the SNc and VTA ROIs, we used a binary mask created from an anatomically specified ROI based on single-subject structural scans (Pauli et al., 2018), but not the structural images of the current sample.

The basolateral and centromedial amygdala ROIs were derived from the CIT atlas, which is in the same standard space as the functional images (Tyszka and Pauli, 2016). The anatomical ROIs of the caudate, palldium and putamen were derived from the Harvard-Oxford Subcortical Atlas (Smith et al., 2004).

To visualize results in our *a priori* ROIs, results were corrected for multiple comparisons with *q* < 0.05, FDR (false-discovery rate) across a merged mask of all ROIs (OFC, Insula, Amygdala, Accumbens, Caudate, Pallidum and Putamen, SNc, VTA and Habenula). To create this mask, we included voxels with 75% or higher probability from each probabilistic subcortical atlas and all voxels from the bilateral Insula and bilateral Orbital Frontal Cortex atlas.

Additionally, we conducted comparisons of mean activity across different conditions in the *a priori* ROIs. When performing ROI analyses, we looked at mean activity in each ROI across subjects, calculated based on the individual subject-level beta images for each condition from the first-level analysis. For tests of ROI activity, *p* < 0.005 (Bonferroni corrected for comparisons across 10 ROIs) was considered significant, and for each test, we report whether it exceeded the Bonferroni correction.

Results that did not exceed the FDR threshold across the mask of all ROIs or survive Bonferroni correction are reported for information only. For visualization purposes for ROI results in the basal ganglia, substantia nigra and habenula, we plot the results at an uncorrected threshold of *p* < 0.001 or *p* < 0.005. An additional result from the habenula ROI is shown at *q* < 0.05, FDR, small volume corrected with a binary mask of the ROI.

## Results

### Behavioral Results: Monetary Reward Task

In order to investigate the behavioral effects of conditioned inhibition, we ran a follow-up behavioral study using monetary rewards to look at how the conditioned inhibition procedure affected reward expectation. This study allowed us to examine the behavioral effects of conditioned inhibition by having participants rate their expectation of reward at the end of each training block. As outlined above, conditioned inhibition occurs if a CI presented concurrently with a CS+ is able to elicit a reduced conditioned response compared to the CS+ alone. To investigate whether there was a behavioral effect, we asked participants to rate how strongly they would expect reward on a continuous rating scale, ranging from No Expectation to Strongest Expectation after viewing the fractal stimulus. This behavioral test revealed that our conditioning procedure was successful, as mean ratings across blocks showed a significantly higher rating for the CS+ than the CS– [*t*(18) = 11.84, *p* < 0.001]. We also found that the ratings for the CS+ when presented concurrently with the Inhibitor were significantly lower than the ratings for the CS+ alone [*t*(18) = 7.07, *p* < 0.001], indicating that the conditioned inhibition procedure had significantly reduced reward expectations, demonstrating conditioned inhibition. See Figure 2 for an illustration of the behavioral ratings for the CS+, CS–, and CS+ paired with the Inhibitor.

### fMRI Results

#### BOLD Responses to Reward Delivery

Based on prior studies, we expected that presentation of the juice reward would lead to activity in sensory regions associated with gustatory sensations, such as the insula, along with regions associated with reward outcomes, including juice rewards, such as the amygdala, OFC, midbrain, and striatum (O’Doherty et al., 2001; Kringelbach et al., 2003; O’Doherty et al., 2003; D’Ardenne et al., 2008; Frank et al., 2008; Metereau and Dreher, 2013; Pauli et al., 2015). We conducted a whole brain analysis to look at the effects of juice reward presentation compared to the neutral control solution. This comparison compared juice presentation (the expected presentation of reward following the CS+) with the presentation of the neutral control solution following each CS– cue. However, there were no significant voxels at *q* < 0.05 across the whole-brain mask.

**FIGURE 3:**
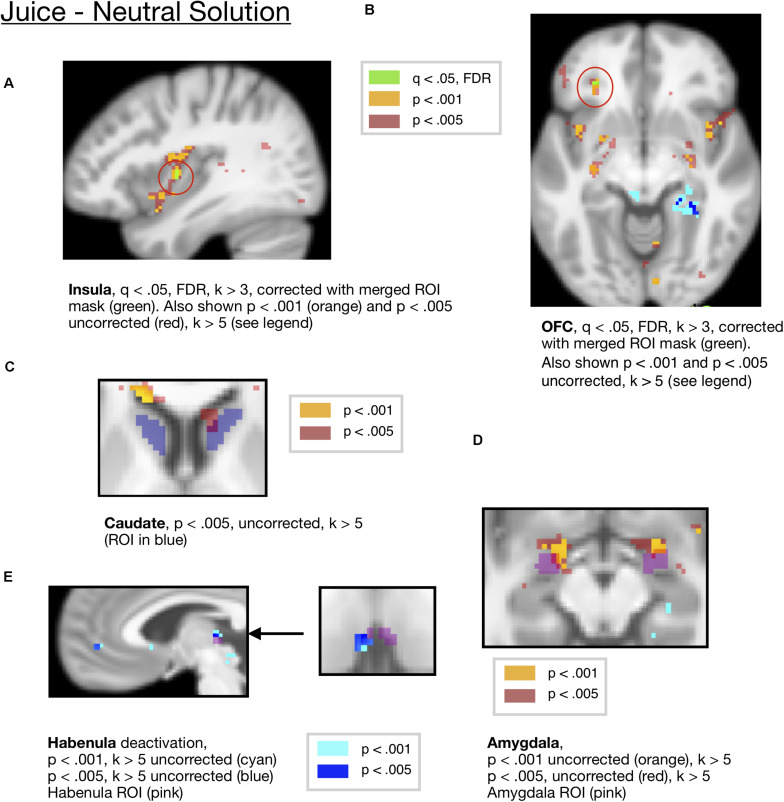
**(A)** Juice compared to the neutral solution showed activity in the Insula ROI (*x* = 38, *y* = –4, *z* = —6, *t* = 6.51, *k* = 3), corrected at FDR *q* < 0.05 within the all-ROI mask. **(B)** Juice compared to neutral solution showed activity in OFC, corrected at FDR *q* < 0.05 with the all-ROI mask. (*x* = –28, *y* = 36, *z* = –8, *t* = 7.94, *k* = 3 voxels). Activity also shown at *p* < 0.001 and *p* < 0.005 for visualization. **(C)** Juice compared to neutral solution showed activity in the caudate ROI (*x* = 10, *y* = 20, *z* = 4, *t* = 7.68, *k* = 19) at *p* < 0.005, uncorrected. **(D)** Juice compared to neutral solution showed activity in the amygdala ROI (*L* = –20,–2,–12, *t* = 20.27, *k* = 34, *R* = 22,0,–14, *t* = 10.4, *k* = 21) at *p* < 0.001 uncorrected. **(E)** Juice compared to neutral solution showed a deactivation in the habenula ROI (*x* = −4, *y* = −24, *z* = 4, *t* = 10.12, *k* = 8) at *p* < 0.001 and *p* < 0.005 (*x* = −4, *y* = −22, *z* = 4, *t* = 10.12, *k* = 14) uncorrected.

We also conducted ROI analyses in a set of *a priori* ROIs including the OFC, amygdala, insula, and striatum. To visualize these results and correct for multiple comparisons across ROIs, we show activity that survived correction across the ROI mask in Figures 3A,B. Activity in the OFC [*x* =−28, *y* = 36, *z* =−8, *t* = 7.94, *k* = 3] and insula ROIs [mm center *x* = 38, *y* =−4, *z* = 6, *t* = 6.51, *k* = 3] for juice compared to neutral solution survived FDR correction at *q* < 0.05 corrected across a mask of all ROIs, as shown in Figures 3A,B. For visualization purposes, we also show activity at *p* < 0.005 and *p* < 0.001 uncorrected in these regions.

These regions are summarized in Table 1, among other key contrasts.

**TABLE 1:** Summary of results across contrasts; regions that survived whole-brain correction, either at whole-brain FDR corrected threshold, or with FDR correction across a mask of all ROIs or *p* < 0.001 or *p* < 0.005 in *a priori* ROIs.

Brain region	*x*	*y*	*z*	*t*	*k*	*p*	Correction	SVC
**Whole-brain, FDR *q* < 0.05**								
**CS+ > CS–**								
Insula	30	26	0	8.31	11	<0.05	FDR	N
Insula	–30	22	4	11.8	5	<0.05	FDR	N
**Omission > Neutral**								
Orbitofrontal Cortex	40	24	10	5.83	8	<0.05	FDR	N
Insula	48	18	–4	6.16	8	<0.05	FDR	N
Middle Temporal Gyrus	56	–42	–4	6.12	6	<0.05	FDR	N
**Inhibitor + Unexpected Reward**								
Putamen	32	–16	–4	7.16	5	<0.05	FDR	N
**Whole-brain, FDR *q* < 0.05, ROI mask**								
**Juice > Neutral**								
Insula	38	–4	6	6.51	3	<0.05	FDR	Y
OFC	–28	36	–8	7.94	3	<0.05	FDR	Y
**CS+ > CS-**								
OFC	–24	18	–20	5.91	8	<0.05	FDR	Y
OFC	–34	20	–20	5.46	8	<0.05	FDR	Y
SNc	8	–14	–12	4.8	1	<0.05	FDR	Y
**CS+ > Inhibitor**								
SNc	12	–18	–12	6.72	1	<0.05	FDR	Y
***p* < 0.001, Uncorrected**								
**Juice > Neutral**								
Amyg (L)	–20	–2	–12	20.27	34	<0.001	Unc.	Y
Amyg (R)	22	0	–14	10.38	21	<0.001	Unc.	Y
***p* < 0.005, Uncorrected**								
**Juice > Neutral**								
Caud	10	20	4	7.68	19	<0.005	Unc.	Y
Caud	12	12	14	7.24	8	<0.005	Unc.	Y
**CS+ > CS–**								
SNc	8	–14	–12	8.85	7	<0.005	Unc.	Y
**Inhibitor > Controls**								
LHb	–2	–24	–2	6.71	11	<0.005	Unc.	Y
Striatum (Inc. Pallidum, Putamen)	20	4	–6	11.29	53	<0.005	Unc.	Y
**FDR *q* < 0.05, SVC**								
**Inhibitor > Controls**								
LHb*	–2	–24	0	3.76	2	<0.05	FDR	Y

*Note that habenula activity for Inhibitor > Controls did not survive correction across the mask of all ROIs, and was small-volume corrected within the habenula mask.

There was activity at a whole-brain uncorrected threshold of *p* < 0.001 in regions expected from prior studies of rewarding outcomes, including the insula, orbitofrontal cortex, basolateral amygdala and putamen for juice compared to the neutral solution, as shown in Supplementary Table S2. Additional results from ROI analyses that did not exceed the Bonferroni-corrected *p*-value are reported in the Supplementary Information. However, we present this for information only, noting that it did not survive correction for multiple comparisons.

We further examined activity to an unexpected reward omission, examining the 25% of trials where the CS+ was unexpectedly followed by the neutral solution compared to neutral solution presentation following control trials (where it was expected). This revealed two peaks in the orbital frontal cortex [*x* = 40, *y* = 24, *z* = –10, *k* = 8, *t* = 5.83] and insula [*x* = 48, *y* = 18, *z* = –4, *k* = 8, *t* = 6.16] surviving FDR correction at *q* < 0.05 correction across the whole brain.

We also conducted analyses to compare the mean activity of voxels in several ROIs for juice reward presentation compared to presentation of the neutral solution, correcting for the number of ROIs used.

An ROI analysis of mean activity averaged across the caudate ROI showed significant activity for juice compared to neutral solution [*p* = 0.0121, Bonferroni corrected *p* = 0.121, *t*(18) = 2.79 mm center *L* = 14,12,10 and *R* = −12,10,10], which did not survive the Bonferroni correction. The activity in the caudate ROI did not survive FDR correction across the mask of ROIs at *q* < 0.05, FDR. There were two peaks within the caudate at an uncorrected threshold of *p* < 0.005, one located within dorsal caudate [mm center = 10,20,4, *k* = 19, *t* = 7.68], and the other in more ventral caudate [mm center = 12,12,14, *k* = 8, *t* = 7.24], shown in Figure 3C. We present this for information only but not for making inference, as the caudate cluster did not survive multiple-comparisons correction.

Additionally, there was significant ROI activity in the central amygdala ROI for the juice reward compared to neutral solution [*p* = 0.0033, Bonferroni corrected *p* = 0.033, *t*(18) = 3.378, mm center *L* = 24,–8,–10, *R* = –24,–10,–12], which survived Bonferroni correction across all ROIs. For visualization purposes, this is shown at an uncorrected threshold of *p* < 0.001 and *p* < 0.005 in Figure 3D.

An ROI analysis of mean activity in the habenula ROI showed a significant deactivation for juice presentation compared to the neutral solution [*p* = 0.0452, *t*(18) = –2.15, mm center *L* = 4,–24,2, *R* = –2,24,2], however, this did not survive the Bonferroni corrected threshold. The activity in the habenula ROI did not survive FDR correction across the mask of ROIs at *q* < 0.05, FDR. For visualization purposes, this is shown at an uncorrected threshold of *p* < 0.001 and *p* < 0.005 in Figure 3E.

The substantia nigra and VTA ROIs did not show significant activity for juice compared to the neutral solution. All other comparisons of ROI activity that did not survive Bonferroni correction are reported in the Supplementary Material Section 1.1, and summarized in Supplementary Table S1. Results from the ROI analysis for the juice compared to neutral solution contrast, along with ROI results from other contrasts, are summarized in Table 1.

#### BOLD Responses to CS Presentations

**FIGURE 4:**
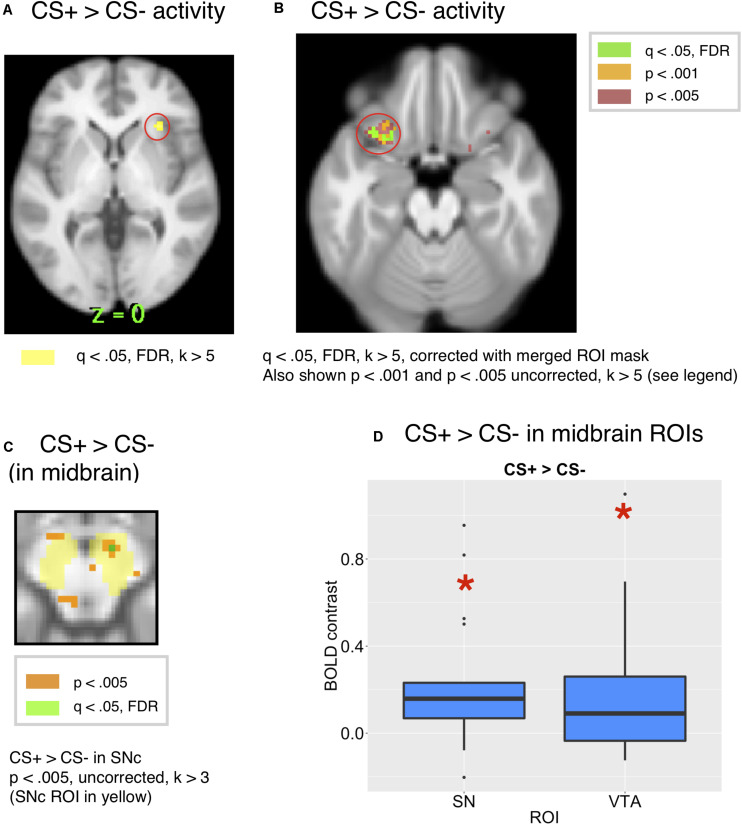
**(A)** Whole-brain activity for the CS+ compared to CS– showed activity in the insula at *q* < 0.05, FDR. **(B)** Comparing the whole brain activity for the CS+ to that for the CS–, there was activity in the OFC (*x* = 24, *y* = 18, *z* = 20, *t* = 5.91, *k* = 8 and *x* = –34, *y* = 20, *z* = 20, *t* = 5,46), corrected across the all-ROI mask. **(C)** CS+ > CS– in the SN/VTA, *p* < 0.005, uncorrected. (peak: 8, –14, –12, *k* = 7, *t* = 8.85). **(D)** The ROI analysis showed significant activity in the SNc [*t*(18) = 3.17, *p* = 0.0053, Bonferroni *p* = 0.053) and VTA [*t*(18) = 2.58, *p* = 0.0189, Bonferroni *p* = 0.189] for the CS+ compared to the CS–.

We expected that a CS associated with reward would increase BOLD signals in the orbitofrontal, insular and ventromedial prefrontal cortical regions (Kim et al., 2011; Diekhof et al., 2012). As expected, we found activity in the bilateral insula for the CS+ compared to the CS– at *q* < 0.05, FDR, *k* > 5, corrected across the whole brain [*x* = 30, *y* = 26, *z* = 0, *t* = 8.31, *k* = 11 and *x* = −30, *y* = 22, *z* = 4, *k* = 5], consistent with other studies that have found activity in the insula for food reward cues (Tang et al., 2012). This is shown in Figure 4A.

Further, we conducted a focused ROI analysis of activity to the CS+ compared to the CS+, correcting across a merged mask of all ROIs. While there was activity in the orbital frontal cortex for a CS+ compared to a CS– [*x* = –28, *y* = 18, *z* = –20, *t* = 10.97, *k* = 26, and *x* = 28, *y* = 18, *z* = –16, *t* = 8.2, *k* = 16], this activity was significant at *p* < 0.001, uncorrected, but did not survive the whole-brain corrected FDR threshold. However, activity in the OFC ROI survived FDR correction at *q* < 0.05, *k* > 5 voxels within the mask of all ROIs [*x* = –24, *y* = 18, *z* = –20, *t* = 5.91, *k* = 8 and *x* = –34, *y* = 20, *z* = –20, *t* = 5.46, *k* = 8], as shown in Figure 4B, along with activity at an uncorrected threshold of *p* < 0.001 and *p* < 0.005 for visualization. Additionally, there were two peaks in the insula at *q* < 0.05, FDR, corrected across the all-ROI mask [*x* = 32, *y* = 24, *z* = 0, *k* = 53, *t* = 8.31 and *x* = −30, *y* = 22, *z* = 4, *k* = 37, *t* = 11.8].

The regions that survived FDR correction across the mask of all ROIs for the CS+ > CS–, along with other contrasts, are summarized in Table 1.

Further peaks from the whole-brain threshold of *p* < 0.001 uncorrected for the CS+ compared to the CS– include insula, thalamus and midbrain as described in Supplementary Table S4. At a threshold of *p* < 0.005 uncorrected, there was a cluster including the striatum (caudate) and extending to the pallidum [*x* = –12, *y* = 8, *z* = 2, *t* = 9.71, *k* = 25] for the CS+ compared to the CS–. However, this did not survive FDR correction across the mask of all ROIs, and is mentioned for information only, noting that it did not survive correction for multiple comparisons.

We expected activity for conditioned stimuli associated with reward in the midbrain, amygdala and striatum (Breiter et al., 2001; O’Doherty et al., 2002, 2006; Pauli et al., 2015). We next conducted an ROI analysis based on prior studies which found responses in the midbrain for predictors of a positive valenced reward (Adcock et al., 2006; O’Doherty et al., 2006; Pauli et al., 2015). As predicted, we found more activity in SNc [*t*(18) = 3.17, Bonferroni corrected *p* = 0.053, *p* = 0.0053, mm center *L* = 8,–18,–14, *R* = –8,–20,–14] and VTA [*t*(18) = 2.58, *p* = 0.0189, Bonferroni corrected *p* = 0.189 mm center = 0,–20,–16] for the CS+ than the CS–, as shown in Figure 4D. While the CS+ > CS– effect did not exceed the Bonferroni corrected *p*-value threshold in the SNc or VTA ROIs, it was just below the margin of significance in the SNc. For visualization purposes, the CS+ > CS– effect in substantia nigra is shown in Figure 4C at *p* < 0.005. However, only a single voxel in this region [*x* = 8, *y* = −14, *z* = −12, *t* = 6.72, *k* = 1] survived correction for multiple comparisons at *q* < 0.05, FDR across the mask of all ROIs. The CS+ > CS– effect was only visible an a lower, uncorrected threshold of *p* < 0.05 in the VTA ROI, which is shown in Supplementary Figure S6 for visualization, but we note that it did not survive correction for multiple comparisons.

There was not significant ROI activity in the amygdala, nucleus accumbens, caudate, pallidum or putamen for the CS+ compared to the CS–.

#### BOLD Responses to the Conditioned Inhibitor

We expected that the CI would recruit activity in regions that have been shown to respond to predictors of reward omissions. However, we are unaware of other fMRI studies using a conditioned inhibition design with rewards, so it is unclear whether the same regions that have been shown to respond to predictors of monetary loss and aversive stimuli also respond to CIs, or predictors of reward omission. A CI has never explicitly been followed by a negative valence outcome, but acquires negative value by reliably signaling a reward omission. Based on computational theories of learning such as TD, Rescorla–Wagner and PVLV (Rescorla, 1969a; Sutton and Barto, 1990; O’Reilly et al., 2007; Mollick et al., 2020), this occurs because the negative reward prediction errors that occur when a predicted reward is unexpectedly omitted cause the CI that predicts reward omission to acquire negative value. We conducted a whole brain analysis for regions responding to the CI compared to control stimuli, but did not find any regions that survived correction for multiple comparisons at *q* < 0.05, FDR.

While there is little research on brain areas that encode CIs in humans [though see Meyer et al. (2019) for a negative valence version], previous studies have shown that predictors of monetary loss are associated with BOLD activity in the insula (Samanez-Larkin et al., 2008).

Consistent with this data, we also saw significantly more mean activity in the bilateral insula ROI for the inhibitor than the control stimuli [*p* = 0.0386, *t*(18) = 2.23, mm center *L* = 38,4,0, *R* = –36,2,0]. However, this activity did not survive correction at FDR *q* < 0.05 across the mask of ROIs or Bonferroni correction for multiple comparisons. In the whole-brain analysis for the inhibitor compared to controls, there was activity in the insula at *p* < 0.005 uncorrected, as shown in Supplementary Figure S2. We present this for information only, noting it did not survive correction for multiple comparisons.

While human fMRI studies have found activity in the habenula to predictors of aversive stimuli (Lawson et al., 2014) as well as aversive outcomes (Hennigan et al., 2015), and negative reward prediction error signals associated with reward omissions (Salas et al., 2010), it is unclear whether the habenula shows activity for predictors of reward omission in humans.

Based on animal studies, we predicted that the habenula would show an increase in activity for the CI, as it showed an increase in activity for a CS that predicted omission of reward, accompanied by a reduction in SN/VTA activity for the Inhibitor (Tobler et al., 2003; Matsumoto and Hikosaka, 2009b). Consistent with this prediction, there was significant activity in the habenula for the CI viewed alone compared to the mean activity for all control stimuli, including the CS– (B), the single stimulus neutral cue (Y–) and the compound stimulus (BY) neutral cue [*t*(18) = 2.22, *p* = 0.0397, Bonferroni corrected *p* = 0.397, mm center *L* = 4,–24,2, *R* = –2,–24,2]. However, this activity for the CI did not survive the Bonferroni corrected threshold.

**FIGURE 5:**
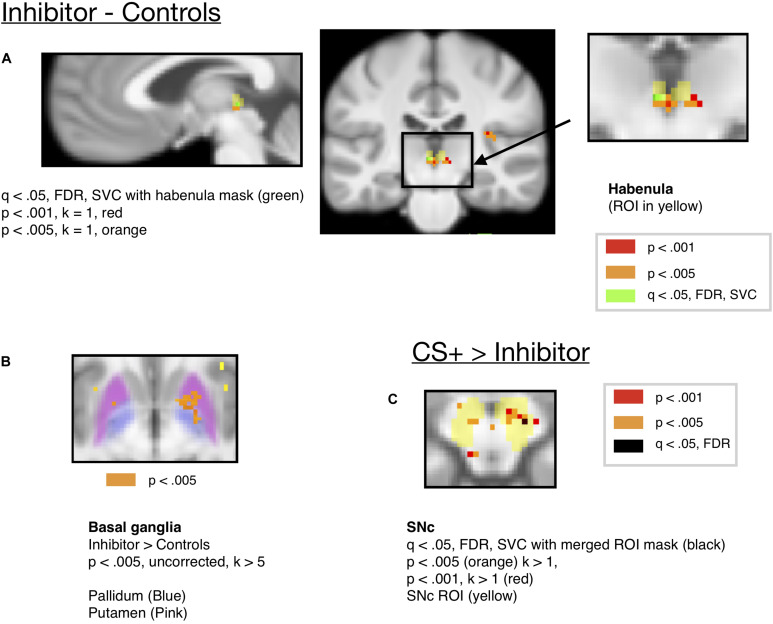
**(A)** Activity for the Conditioned Inhibitor compared to the control stimuli was significant in the habenula at *p* < 0.005 (*x* = –2, *y* = –24, *z* = –2, *t* = 6.71, *k* = 11). **(B)** Activity in the basal ganglia ROIs (Pallidum and Putamen) for the Inhibitor > Controls at *p* < 0.005. **(C)** A contrast comparing activity for the CS+ to the Inhibitor showed activity in the SNc at *p* < 0.005. (*x* = 8, *y* = –16, *z* = –12, *k* = 11, *t* = 12.35).

Further, this was not significant when the inhibitor was compared to only the neutral (Y–) control (consisting of a single cue shown at a similar rate to the inhibitor) that was always followed by the neutral solution [*t*(18) = 1.119, *p* = .2779, Bonferroni corrected > 1, mm center *L* = 4,–24,2, *R* = –2,–24,2]. Habenula activity for the CI compared to the controls is shown at an uncorrected threshold of *p* < 0.005 and *p* < 0.001 in Figure 5A, along with 2 voxels surviving FDR correction at *q* < 0.05 [*x* = –2, *y* = –24, *z* = 0, *t* = 3.76, *k* = 2], small-volume corrected with the habenula mask. However, this region did not appear when FDR correction was done across the mask of all ROIs, and therefore we strongly qualify this result, which awaits further replication before inference can be made.

**FIGURE 6:**
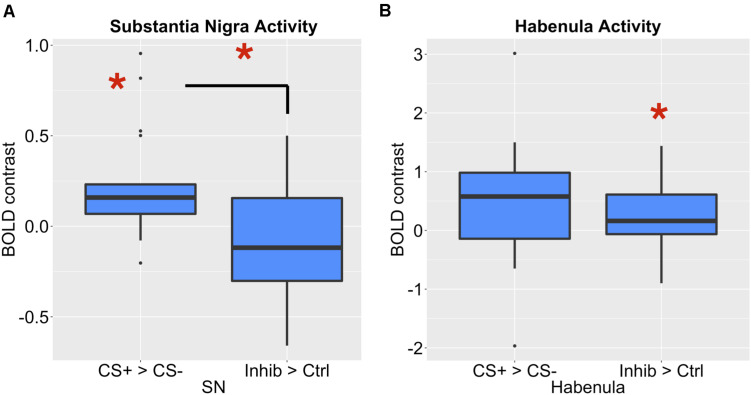
**(A)** The SNc showed a significant increase for a CS+ compared to a CS–, but not for an Inhibitor compared to a control cue. There was a significant difference between these effects. **(B)** The LHb showed a significant increase for an Inhibitor compared to a control cue, but not a CS compared to a CS–.

To compare the role of the habenula and substantia nigra in our learning task, we compared activity in both ROIs, as shown in Figure 6. Consistent with the hypothesis that the substantia nigra encodes positive valence, activity in the substantia nigra increased for the CS+ paired with reward compared to the CS–, but not for the Inhibitor compared to control stimuli. We further found that there was significantly more activity in the substantia nigra for the CS+ > CS– effect than Inhibitor > Control comparison in the substantia nigra [*p* = 0.003452, Bonferroni corrected *p* = 0.03452, *t* = 3.13]. Further, limited evidence pointed towards the habenula encoding negative valence, as it significantly increased for an Inhibitor paired with a reward omission, but not the positively valenced CS+, though this comparison did not survive Bonferroni correction. However, there was not a significant difference between the Inhibitor > Control and the CS+ > CS– effect in the habenula [*p* = 0.5281, Bonferroni corrected p > 1, *t* = –0.6371].

Along with the habenula, we also expected that regions of the basal ganglia would respond to the CI associated with reward omissions. The ventral striatum has been shown to be activated by predictors of aversive stimuli (Jensen et al., 2003), and a cue associated with monetary loss activated a more posterior region of the ventral striatum (Seymour et al., 2007a). Further, animal studies have shown that pallidum communicates aversive expectations to habenula (Hong and Hikosaka, 2008), and studies showed that basal ganglia stimulation influenced habenula activity (Hong and Hikosaka, 2013). There was activity in the putamen region of striatum [*x* = 20, *y* = 4, *z* = –6, *k* = 53, *t*(18) = 11.29], which extended into the pallidum, for the inhibitor compared to control stimuli at *p* < 0.005, uncorrected, as shown in Figure 5B. We provide this for information only, but not for making inference as it did not survive correction for multiple comparisons. There was an increase in, the mean ROI activity in the pallidum and putamen ROIs for the inhibitor compared to control stimulus but this did not reach the significance threshold (see Supplementary Table S1 and Supplementary Section 1.1).

Additionally, shown in Figure 5C, we ran a contrast comparing activity for the CS+ to that for the Inhibitor, which showed activity in the substantia nigra at *p* < 0.005 uncorrected, but this activity did not survive correction for multiple comparisons. Only a single voxel in the SNc survived correction across the mask of all ROIs at *q* < 0.05, FDR [*x* = 12, *y* = –18, *z* = 12, *k* = 1, *t* = 6.72]. Further, an ROI analysis of mean ROI activity in the SNc showed more activity for the CS+ than the CI [*p* = 0.01 uncorrected, Bonferroni corrected *p* = 0.1, *t*(18) = 2.88, mm center *L* = 8,–18,–14, *R* = –8,–20,–14], but this did not survive Bonferroni correction for the number of ROIs.

If the inhibitor acquired a negative association, there should be a prediction error when the inhibitor is paired with a reward, resulting in activity in dopamine regions. However, an analysis looking at the mean activity in the VTA, SNc or Accumbens ROIs for the inhibitor followed by a reward did not show significant activity in the trials where the Inhibitor was followed by an unexpected reward.

There was a significant increase in activity in the putamen ROI during taste presentation when the inhibitor was unexpectedly followed by reward which survived whole-brain FDR correction at *q* < 0.05, FDR [*x* = 32, *y* = –16, *z* = –4, *t* = 7.16, *k* = 5]. Further, an ROI analysis of mean activity in the putamen ROI showed an increase in the putamen ROI during taste presentation when the inhibitor was unexpectedly followed by reward, compared to when the inhibitor was followed by a neutral solution [*p* = 0.0417, *t*(18) = 2.19, mm center *L* = 26,2,0, *R* = –26,2,0], but this did not exceed the threshold for Bonferroni correction.

An additional, more sensitive test of conditioned inhibition may be the comparison of responses to the inhibitor followed by reward to the control stimulus followed by reward, as the Tobler et al. (2003) study showed a larger response to the inhibitor followed by reward than the control stimulus followed by reward. This may reflect a greater prediction error resulting from the unexpected reward presentation following the CI compared to the control stimulus. Greater prediction errors when the inhibitor is followed by reward may occur because computational models such as the Rescorla–Wagner model suggest that the inhibitor acquired negative value through the conditioned inhibition procedure, compared to the control stimulus which has no inhibitory association and thus a smaller prediction error when unexpectedly followed by reward. However, when we compared these two conditions, there was not a significant difference in putamen mean ROI activity when inhibitor was unexpectedly followed by reward compared to when the control stimulus was unexpectedly followed by reward [*p* = 0.3305, Bonferroni corrected *p* > 1, *t* = 1.00]. The regions that survived whole brain correction for the Inhibitor compared to Controls and the Inhibition test are summarized in Table 1. Notably, only the signal for the inhibitor paired with reward survived correction for multiple comparisons across the whole brain.

Based on our discussion of the potential of conditioned inhibition to dissociate between representations of associations of a CS+ with reward and the representation of an inhibitor with reward omissions, we compared the activity for the CS+ and Inhibitor to that of the Inhibitor viewed alone. This should reveal regions that selectively reflected the associations of the CS+ with reward. Three clusters in visual cortex, including the lingual gyrus, showed more activity for the CS+ and Inhibitor compared to the Inhibitor alone, as described in Supplementary Table S7. However, there were also important visual differences between the CS+ and Inhibitor, as the CS+ was represented by a single visual cue and the Inhibitor was represented by two visual cues. Therefore, it is difficult to interpret whether the visual cortical regions reported above reflect visual differences between the cues or the association of the CS+ with reward. Further, it is possible that activity in this region may also reflect the conjunction of the combined stimulus consisting of the CS+ and the Inhibitor.

## Discussion

Recent research has suggested that the lateral habenula drives dopamine dips for aversive stimuli and reward omissions, so we expected a selective activation of the habenula for the CI, paired with a reduction in SN/VTA activity. Consistent with these predictions, we found significant activity at an uncorrected threshold in habenula for a CI associated with reward omission compared to the mean of all control stimuli (but not when compared to the second control stimulus). However, the activity in the habenula for the CI did not survive FDR correction across the mask of all ROIs, and the test of mean ROI activity for the inhibitor compared to controls in habenula did not exceed the Bonferroni-corrected *p*-value threshold, and thus should not be strongly interpreted. While other studies have found activity in habenula for predictors of aversive outcomes, such as shock (Lawson et al., 2014), or an aversive bitter juice outcome (Hennigan et al., 2015), and one study found habenula activity during the omission of an expected reward (Salas et al., 2010), none have shown that a reward omission stimulus, or CI, drives habenula activity in humans. The habenula signals we found for a CI are consistent with a recent animal study (Laurent et al., 2017), which found that projections from the habenula to the RMTg, the tail region of the VTA, which sends inhibitory connections to dopamine neurons (Bourdy and Barrot, 2012) were crucial for the effects of a CI on choice. One limitation of our study is that, due to the resolution of standard fMRI data, our use of smoothing, and the small size of habenula (Lawson et al., 2013), we cannot differentiate medial from lateral habenula. This limits our ability to directly relate to the lateral habenula signals observed in animal studies. Further, while there was activity in the habenula for the inhibitor compared to the mean of all control stimuli, this was not significant when the inhibitor was compared to a single control stimulus, and the Inhibitor > Controls effect was not significantly different than the CS+ > CS– effect. These may speak to a lack of power due to our small sample size, and the small effect size of the habenula findings await future replication before inferences can be made.

Though increased activity in the habenula is associated with a dip, or pause in tonic dopamine firing (Matsumoto and Hikosaka, 2009b), we did not see a significant reduction in SN/VTA activity during presentation of the CI. While we did not see a significant decrease in substantia nigra or VTA activity for the CI compared to a neutral CS–, few studies have actually shown a significant decrease in BOLD in dopaminergic areas during a negative reward prediction error. For example, D’Ardenne et al. (2008) did not see a significant decrease in SN/VTA activity when an expected reward was omitted, and Rutledge et al. (2010) similarly did not find signals consistent with reward prediction error encoding in these midbrain areas.

One potential reason that we did not see significant reductions in BOLD signals for the CI could be related to the physiology of the midbrain dopamine system. For example, inhibitory synaptic input has been shown to increase BOLD signals (Logothetis, 2008), and it is possible that inhibitory signals during reward omissions are conveyed from the lateral habenula to GABAergic neurons in the RMTg (which inhibit the SN/VTA). Further, these inhibitory neurons are spatially close to dopaminergic neurons and may not be spatially resolvable with the resolution of fMRI (Düzel et al., 2009). Such signals could potentially explain cases where SN/VTA activity increased for an aversive stimulus, for example, Pauli et al. (2015) found that neurons in the SN showed an aversive value signal, and Hennigan et al. (2015) found activation of the SN for a shock stimulus. Another potential explanation is that the inhibitor signaled the omission of the expected reward, which was a salient event, and activity in dopamine neurons as well as BOLD signals in human studies have been associated with salient and novel events (Horvitz, 2000; Matsumoto and Hikosaka, 2009a; Bromberg-Martin et al., 2010b; Krebs et al., 2011; Richter et al., 2020).

We also replicated previous studies, which showed activation in the SN/VTA area for a rewarding outcome, and studies showing activity in the SN/VTA area for a CS+ paired with reward (O’Doherty et al., 2002). While we expected signals in the striatum and amygdala during anticipation of the juice reward (O’Doherty et al., 2002), we did not see significant amygdala signals for the CS+ compared to CS–, and the striatal activity during reward anticipation did not survive the whole-brain corrected threshold.

While some studies have found activations in ventral striatum for pleasant taste presentation (Frank et al., 2008), other studies found more dorsal regions of striatum (O’Doherty et al., 2001; McClure et al., 2003; Frank et al., 2012; Hennigan et al., 2015), or did not observe striatal activity for taste presentation (O’Doherty et al., 2002). We found that regions of the basal ganglia were involved in learning about reward, as the caudate showed activity during presentation of the juice reward compared to a neutral solution, consistent with other studies (O’Doherty et al., 2002), but this activity did not survive FDR correction across the mask of ROIs. We also observed activity in the putamen for juice compared to the neutral solution, but this did not survive correction for multiple comparisons. Activity in the dorsal striatum, including the dorsal caudate, has been correlated with pleasantness ratings (Small et al., 2003), and putamen activity has also been associated with the subjective feeling of appetite (Porubská et al., 2006).

We also predicted that regions of the basal ganglia send signals to the lateral habenula encoding the level of reward expectation, allowing it to drive a dopamine dip if an expected reward is not received. While there was activity pallidum and putamen for the inhibitor compared to a control stimulus that was significant at a whole-brain uncorrected threshold, this activity did not survive correction for multiple comparisons and should not be strongly interpreted. As the behavioral ratings from the Monetary reward task demonstrated that the inhibitor in that study acquired negative value, and the imaging study found that the inhibitor led to activity in the pallidum, this is consistent with another study that observed pallidal activity increasing with the negative value of a shock cue (Lawson et al., 2014). While animal studies have shown that the pallidum encodes both positive and negatively valenced outcomes (Tachibana and Hikosaka, 2012) and signals about reward and punishment pass through the globus pallidus border region to drive activity in the habenula (Hong and Hikosaka, 2008), future studies are needed to understand how these computations are reflected in BOLD signals during reward omission learning, particularly given that the striatal peaks for the inhibitor did not survive correction for multiple comparisons. As with the habenula results, the striatal peaks for the inhibitor await further replication before inferences can be made.

While we also saw a non-significant increase in BOLD activity for the CS+ in the habenula, such differences could potentially be explained by the complexities of mapping neuronal spiking in this region to BOLD signals, and reduced power due to the sample size. Several studies have shown that a CS+ associated with reward decreases neural firing in habenula neurons compared to cues associated with reward omissions (Matsumoto and Hikosaka, 2009b; Bromberg-Martin et al., 2010a). Further, stimulating the output pathway from the habenula led to a decrease in motivational salience to a CS+, indexed by approach behaviors (Danna et al., 2013), while decreasing habenula output lead to an increase in motivational salience, consistent with the idea that activity in habenula projection neurons decreases for reward cues. However, as discussed in our interpretation of midbrain signals, inhibitory synaptic input has been shown to increase BOLD signals (Logothetis, 2008), and in some cases, inhibitory neurotransmission may also lead to increases in metabolic activity that could increase BOLD. If the reward CS+ led to activity in inhibitory input regions projecting to habenula such as the basal ganglia or pallidum (Hong and Hikosaka, 2008, 2013), or inhibitory neurotransmission in habenula neurons inhibited by reward led to an increase in metabolic activity, this could potentially cause increases in BOLD signals to a CS+. Additionally, Bromberg-Martin et al. (2010a) showed increases in habenula activity to both appetitive and aversive cues at the start of a trial, though these same neurons clearly differentiated between a CS+ and the CS– associated with no reward during other parts of the trial.

Further, we observed activity in the putamen surviving correction for multiple comparisons when the CI was unexpectedly followed by a juice reward in the inhibition test at the end of the experiment. This may reflect a prediction error if the conditioned inhibition procedure caused the inhibitor to acquire negative value, consistent with other studies that have found putamen regions respond to prediction error signals (O’Doherty et al., 2003; Seymour et al., 2004). However, when we conducted an additional test comparing the magnitude of putamen ROI signals for the CI unexpectedly followed by reward compared to the Control stimulus followed by reward, we did not find a significant difference, even though the Tobler et al. (2003) study observed a stronger response in dopamine neurons to the CI followed by reward that the control stimulus followed by reward. This may be related to a lack of temporal resolution in our study, as the cues occurred 2 seconds before the responses to outcomes and may have been difficult to resolve from the outcome activity. In addition, by a prediction error encoding account, responses to the cues may have driven the opposite response, with the inhibitor resulting in less activity than the control stimulus.

Along with subcortical regions, we found that the orbital frontal cortex and anterior insula showed involvement in the reward learning task. We replicated prior studies showing activation of the anterior insula for taste stimuli (Nitschke et al., 2006; O’Doherty et al., 2006; Frank et al., 2012). We also observed activity in orbitofrontal cortex for the receipt of the taste stimulus, consistent with other studies (O’Doherty et al., 2001; O’Doherty et al., 2002). Further, we saw activity surviving the whole-brain corrected threshold in the orbitofrontal cortex for a CS associated with a reward, consistent with other studies that have shown activity in orbitofrontal cortex for a CS that predicted reward presentation (Gottfried et al., 2002; Kim et al., 2006). We further saw a signal in the orbital frontal cortex for the negative reward prediction error condition resulting when the CS+ was unexpectedly followed by neutral solution. This finding is consistent with animal data which has shown that the orbital frontal cortex may be particularly important for driving dopamine dip signals for worse than expected outcomes, as dopamine neurons no longer showed that a reduction in firing for an unexpected reward omission when OFC was lesioned (Takahashi et al., 2011). Additionally, a study applying conditioned inhibition in a negatively valanced domain found that children with anxiety disorders represented safety signals (CIs of fear) differently in the vmPFC than children without anxiety (Harrewijn et al., 2020).

We also observed activity for the CI in the anterior insula, but only at a whole-brain uncorrected threshold. This is interesting due to other papers which have suggested a role for the insula in safety signal processing in the aversive domain (Christianson et al., 2008). Activity in the insula has also been related to loss anticipation, as it increases to predictors of loss (Samanez-Larkin et al., 2008) and loss aversion in decision making (Fukunaga et al., 2012), and is related to individual differences in avoidance learning (Paulus et al., 2003). As the CS+ also showed activity in the insula surviving whole-brain correction, there was not selective activation in this region for the inhibitor, and insula has also been associated with positive valence food and drug cues (Tang et al., 2012). The increases in insula activity we saw for both the positively valenced CS+ and the negatively valenced CI are in agreement with papers that have found evidence of “salience” or an unsigned prediction error in the insula (Rutledge et al., 2010). Notably, activity in the insula also showed a significant increase surviving whole-brain correction during trials where the CS+ was unexpectedly followed by the neutral solution, trials which lead to negative reward prediction errors.

Conditioned inhibition provides an interesting way to examine the functioning of the dopamine system and can be used to look at how different brain regions are involved in learning about a CI that predicts not getting reward. It allows comparing the brain activity for stimuli associated with reward predictors to those associated with reward omissions and is interesting for examining how the subcortical areas projecting to the dopamine system, which have been primarily studied in animal learning studies, translate to humans in an fMRI task. Additionally, understanding the brain areas that drive this frustration signal for reward omissions can be translated to understand how disorders that involve persistent negative predictions, such as depression, may involve distortions in these systems. For example, recent research suggests that punishment prediction errors in the lateral habenula correlates with symptoms of depression (Kumar et al., 2018), and future studies could examine whether this relationship extends to reward omission cues. Generally speaking, the neural mechanisms of disappointment or frustration signals involved in conditioned inhibition are understudied relative to rewards, but further understanding of these signals has great translational and clinical relevance; for example, recent animal data indicates that cocaine use impairs the ability of dopamine neurons to suppress firing during omission of an expected reward (Takahashi et al., 2019), and recent human studies have found changes in negative reward prediction error signals in cocaine addiction (Parvaz et al., 2015).

## Data Availability Statement

The datasets presented in this study can be found in online repositories. The names of the repository/repositories and accession number(s) can be found below: all data used for the ROI analysis figures and behavioral rating data can be found in the Open Science Framework: https://osf.io/njbmf/. Neuroimaging data used for the figures of second-level analysis results can be found on Neurovault: https://neurovault.org/collections/8676.

## Ethics Statement

The studies involving human participants were reviewed and approved by Institutional Review Board of the University of Colorado, Boulder. The participants provided their written informed consent to participate in this study.

## Author Contributions

JM, TW, RO’R, and GF contributed to the conception and design of the study. JM performed the statistical analyses of the data. AK, LC, KK, TH, TW, GF, and RO’R contributed to interpretation and discussion of neuroimaging and behavioral results. JM wrote the first draft of the manuscript. All the authors contributed to manuscript revision, read, and approved the submitted version.

## Conflict of Interest

RO’R is CSO and JM, TH, and KK are researchers at eCortex, Inc., Boulder, CO, United States, which may derive indirect benefit from the work presented here. The remaining authors declare that the research was conducted in the absence of any commercial or financial relationships that could be construed as a potential conflict of interest.

## Footnotes

1 https://github.com/canlab/
