# Supplementary material for: The Neural Correlates of Cued Reward Omission
Source: Front Hum Neurosci. 2021 Feb 11;15:615313. doi: 10.3389/fnhum.2021.615313 (PMC7928384; doi:10.3389/fnhum.2021.615313)

Supplementary Material

# Supplementary Data

**Table of contents:**

**Section 1: ROI analyses**

Table S1: Summary of ROI analysis activity

Section 1.1: Supplementary ROI analyses

**Section 2: Whole-brain results:**

Table S2: Table of whole-brain activity for the Juice > Neutral Solution contrast (p < .001 uncorrected)

Table S3: Table of whole-brain activity for Inhibitor > Controls contrast (p < .001 uncorrected)

Table S4: Table of whole-brain activity for CS+ > CS- contrast (p < .001 uncorrected)

Table S5: Table of whole-brain activity for CS+ > CS- contrast (p < .05, FDR)

Table S6: Table of whole-brain activity for Unexpected Reward Omission > Neutral Solution contrast (p < .001 uncorrected)

Table S7: Table of whole-brain activity for CS+ and Inhibitor > Inhibitor contrast (p < .05, FDR)

Table S8: Table of trial types and reward contingencies in each block

**Figures**:

Figure S1: Figure of whole-brain activity for the Juice > Neutral Solution contrast

Figure S2: Figure of whole-brain activity for the Inhibitor > Controls contrast

Figure S3: Figure of whole-brain activity for the CS+ > CS- contrast

Figure S4: Figure of whole-brain activity for Unexpected Reward Omission > Neutral Solution contrast

Figure S5: Rating scale used for reward expectations

Figure S6: Additional figure of CS+ > CS- in VTA and SNc ROIs

## Section 1: ROI analyses:

| **ROI analysis: Mean activity in ROIs** | | |  |  |  |
| --- | --- | --- | --- | --- | --- |
|  |  |  |  |  |  |
| **Juice > Controls** | Coordinates (mm center) | |  | p | T |
| CeA** | -24 | -10 | -12 | 0.0033 | 3.38 |
|  | 24 | -8 | -10 |  |  |
|  |  |  |  |  |  |
| Pallidum | 20 | -4 | -2 | 0.184 | 1.38 |
|  | -20 | -4 | -2 |  |  |
| Caudate* | 14 | 12 | 10 | 0.0121 | 2.79 |
|  | -12 | 10 | 10 |  |  |
| Insula* | 38 | 4 | 0 | 0.0328 | 2.31 |
|  | -36 | 2 | 0 |  |  |
| OFC | -30 | 24 | -16 | 0.0756 | 1.88 |
|  | 30 | -24 | -16 |  |  |
| **Controls > Juice** |  |  |  |  |  |
| Lateral Habenula* | 4 | -24 | 2 | 0.0452 | -2.15 |
|  | -2 | 24 | 2 |  |  |
| **CS+ > CS-** |  |  |  |  |  |
| Insula * | 38 | 4 | 0 | 0.0101 | 2.87 |
|  | -36 | 2 | 0 |  |  |
| OFC * | -30 | 24 | -16 | 0.029 | 2.37 |
|  | 30 | -24 | -16 |  |  |
| SNc * | 8 | -18 | -14 | 0.0053 | 3.17 |
|  | -8 | -20 | -14 |  |  |
| VTA * | 0 | 20 | -16 | 0.0189 | 2.58 |
| **CS+ > CS- (1st block)** | |  |  |  |  |
| Right Caudate | 14 | 12 | 10 | 0.0683 | 1.94 |
|  |  |  |  |  |  |
| **Inhibitor > Controls** | |  |  |  |  |
| Insula * | 38 | 4 | 0 | 0.0386 | 2.23 |
|  | -36 | 2 | 0 |  |  |
| Lateral Habenula * | 4 | -24 | 2 | 0.0397 | 2.22 |
|  | -2 | -24 | 2 |  |  |
| Pallidum | 20 | -4 | -2 | .124 | 1.61 |
|  | -20 | -4 | -2 |  |  |
| Putamen | 26 | 2 | 0 | .1683 | 1.44 |
|  | -26 | 2 | 0 |  |  |
| **Inhibitor > Control 2** | |  |  |  |  |
| Lateral Habenula * | 4 | -24 | 2 | 0.27789 | 1.119 |
|  | -2 | -24 | 2 |  |  |
| **CS+ > Inhibitor** |  |  |  |  |  |
| SNc * | 8 | -18 | -14 | 0.01 | 2.88 |
|  | -8 | -20 | -14 |  |  |
| **Inhibitor + Reward > Inhibitor + Control** |  |  |  |  |  |
| Putamen * | 26 | 2 | 0 | 0.0417 | 2.19 |
|  | -26 | 2 | 0 |  |  |
|  |  |  |  |  |  |
| **Inhibitor + Reward > Control + Reward** | | |  |  |  |
| Putamen | 26 | 2 | 0 | 0.3305 | 1 |
|  | -26 | 2 | 0 |  |  |
| * = Significant at p < .05 (uncorrected), ** = Significant at Bonferroni corrected threshold p < .005 | | | | | |

**Table S1.** Summary of ROI analysis activity in the conditioned inhibition experiment

**Section 1.1: Supplementary ROI analyses**

Here we report additional analyses of mean activity in ROIs that are not reported in the main text, as they did not exceed the Bonferroni-corrected threshold of p < .005 (correcting for the 10 ROIs used).

Juice > Neutral Solution:

An ROI analysis of mean activity in bilateral insula showed significant activity for juice reward compared to neutral solution [p = .0328, t(18)=2.31, mm center L=38,4,0, R=-36,2,0], but only marginally significant activity in bilateral OFC [p = .0756, t(18)=1.88, mm center L=-30,24,-16, R=30,-24,-16] and a non-significant increase in activity in the pallidum ROI [mm center L=-20,-4,-2, mm center R=20,-4,-2, p = .184, t(18)=1.38].

CS+ > CS-

There was significantly more mean ROI activity in the insula for the CS+ compared to the CS- [p = .0101, t=2.87, mm center L=38,4,0, R=-36,2,0]. The ROI analysis showed significantly more activity in the bilateral OFC for the CS+ compared to the CS- [p = .029, t(18)=2.37,mm center L=-30,24,-16, R=30-,24,-16]. However, neither of these tests exceeded the Bonferroni corrected threshold. However, activity in these regions survived FDR correction across the mask of all ROIs.

CS+ > CS-: First conditioning block:

There was a marginally significant increase in mean ROI activity in the right caudate ROI for the CS+ compared to the CS- in the first conditioning block [p= .0683, t(18)=1.94, mm center=14, 12,10]. This did not survive FDR correction across the mask of all ROIs. This survived small-volume correction in the caudate ROI [x=10,y=12,z=0, k=6, t=15.15], but did not survive FDR correction across the mask of all ROIs.

Inhibitor > Controls:

There was an increase in mean ROI activity in the pallidum for the Inhibitor compared to control stimuli, but this did not reach the threshold for significance [p = .124, t(18)=1.61, mm center L=20,-4,-2, R=-20,-4,-2]. Similarly, the putamen also showed an increase in mean ROI activity for the Inhibitor compared to control stimuli which did not reach the significance threshold [p = .1683, t(18)=1.44, mm center L=26,2,0, R=-26,2,0].

**Section 2: Tables of brain activity:**

| Region | x | y | z | Cluster size | T-statistic |
| --- | --- | --- | --- | --- | --- |
| **Juice > Neutral Solution** |  |  |  | (voxels) |  |
| L Temporal Pole | -24 | 4 | -20 | 6 | 10.85 |
| L Amygdala | -20 | -2 | -12 | 34 | 20.27 |
| R Frontal Orbital Cortex | 20 | 32 | -16 | 6 | 8.47 |
| R Amygdala | 22 | 0 | -14 | 21 | 10.38 |
| R Insula | 36 | 8 | -16 | 5 | 9.61 |
| Cerebellum | 8 | -62 | -10 | 8 | 10.84 |
| Putamen | 28 | -10 | -10 | 11 | 9.06 |
| Lingual gyrus | 6 | -82 | -8 | 5 | 10.49 |
| R Insula | 40 | 4 | -4 | 59 | 15.11 |
| Frontal Orbital Cortex | -30 | 34 | -8 | 10 | 12.43 |
| L Putamen | -28 | -16 | -6 | 25 | 20.78 |
| L Insula | -40 | 8 | -8 | 12 | 9.04 |
| Lateral ventricle | -16 | 28 | 6 | 17 | 8.98 |
| Frontal pole | -56 | 44 | 6 | 9 | 8.98 |
| Central opercular cortex | -38 | -10 | 14 | 22 | 9.87 |
| R Precentral gyrus | 62 | 2 | 14 | 54 | 13.64 |
| L Central Opercular Cortex | -50 | -6 | 14 | 13 | 10.43 |
| Postcentral gyrus | -60 | -6 | 16 | 23 | 12.8 |
| R Central Opercular Cortex | 38 | -6 | 18 | 43 | 10.73 |
| **Neutral Solution > Juice** |  |  |  |  |  |
| R Planum Polare | 46 | -8 | -12 | 7 | 12.28 |
| R Parahippocampal gyrus | 22 | -34 | -10 | 7 | 8.65 |
| Lingual gyrus | 30 | -38 | -6 | 17 | 11.04 |
| Posterior cingulate | 20 | -40 | -4 | 6 | 10.19 |
| R Paracingulate gyrus | 8 | 42 | -4 | 21 | 10.74 |
| L Paracingulate Gyrus | -4 | 48 | -2 | 5 | 8.36 |
| R Posterior Cingulate | 12 | -46 | 4 | 7 | 10.48 |
| White matter | -20 | -44 | 4 | 5 | 9.17 |
| L Habenula | -4 | -24 | 4 | 8 | 10.12 |
| Frontal pole | 2 | 64 | 4 | 10 | 8.32 |
| Posterior cingulate | 6 | -48 | 10 | 8 | 15.52 |
| Precuneus Cortex | 0 | -58 | 18 | 5 | 8.57 |

**Table S2.** Juice > Neutral Solution during delivery, p < .001, uncorrected


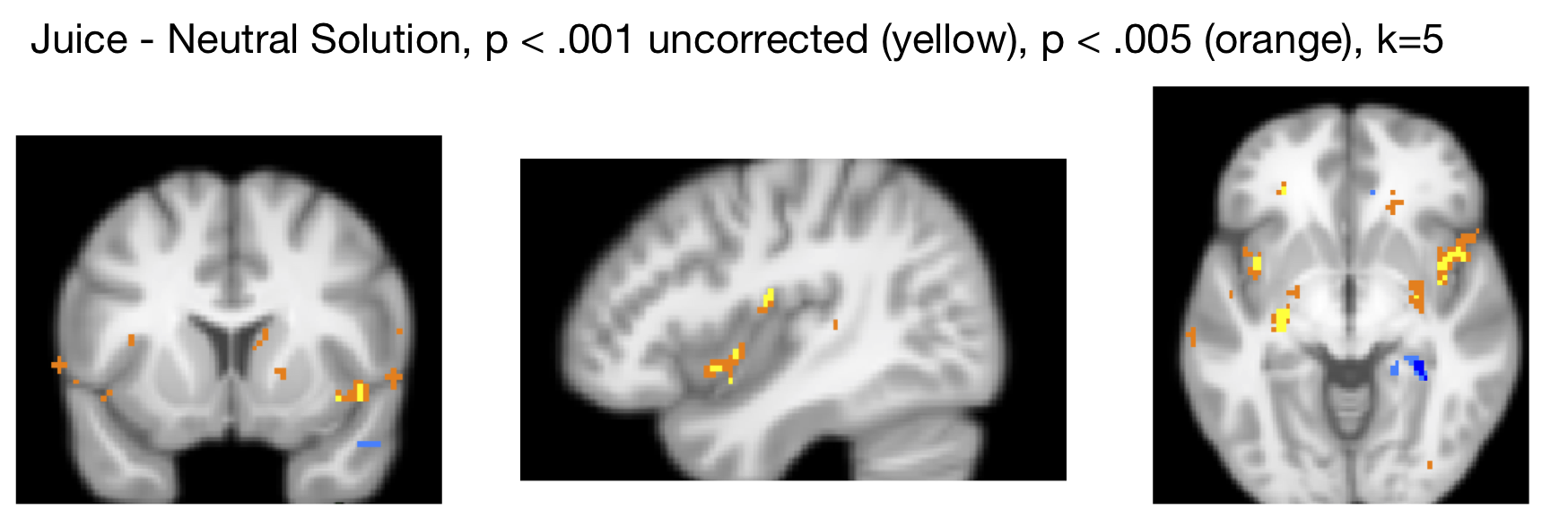


**Figure S1.** Whole brain activity for Juice > Neutral Solution during delivery p < .001, uncorrected (yellow), p < .005 (orange), k = 5


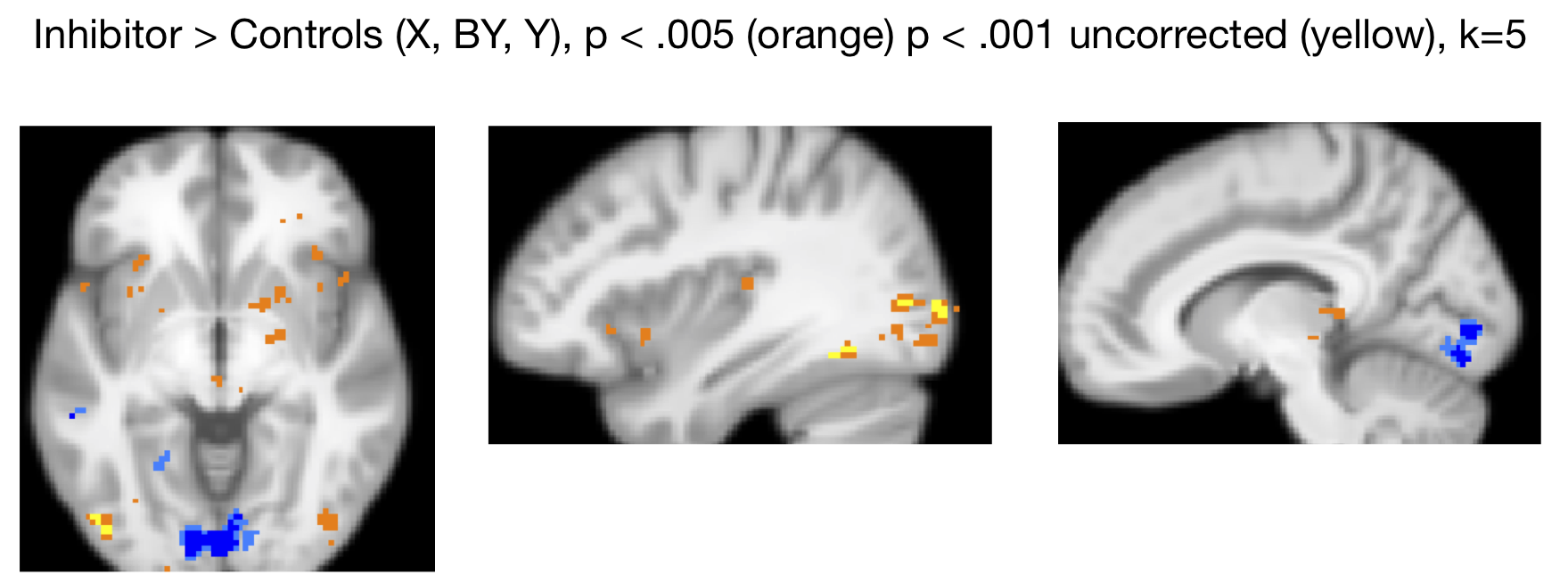


**Figure S2.** Inhibitor > Controls (Y-,BY-,B-), p <.001, uncorrected, k=5

| Region | x | y | z | Cluster size | T-statistic |
| --- | --- | --- | --- | --- | --- |
| **CS+ > CS-** |  |  |  | (voxels) |  |
| Left Orbital Frontal Cortex | -28 | 18 | -20 | 26 | 10.97 |
| Left Orbital Frontal Cortex | -28 | 24 | -20 | 7 | 10.78 |
| Right Orbital Frontal Cortex | 28 | 18 | -16 | 16 | 8.2 |
| Midbrain | 2 | -30 | -6 | 6 | 10.09 |
| Right Middle Temporal Gyrus | 58 | -22 | -6 | 7 | 10.57 |
| Right Insula | 32 | 24 | 0 | 66 | 15.77 |
| Left Insula | -30 | 22 | 4 | 55 | 21.14 |
| Intracalcarine Cortex | -14 | -68 | 6 | 7 | 11.31 |
| Right Thalamus | 8 | -10 | 8 | 6 | 9.34 |
| **CS- > CS+** |  |  |  |  |  |
| White matter | -44 | -20 | -16 | 5 | 9.18 |
| Occpital Pole | 24 | -96 | -8 | 6 | 7.59 |
| White matter | -16 | 32 | 0 | 6 | 8.73 |
| Right Superior Temporal Gyrus | 66 | -22 | 4 | 7 | 13.78 |
| Planum Temporale | 64 | -14 | 4 | 8 | 12 |
| Outside of brain | 64 | 28 | 4 | 8 | 9.05 |
| Heschl's Gyrus | -54 | -14 | 6 | 12 | 12.17 |
| White matter | -10 | 26 | 4 | 9 | 10.39 |
| White matter | -4 | -36 | 10 | 12 | 10.39 |
| Left Superior Temporal Gyrus | -66 | -40 | 12 | 14 | 9.23 |
| Right Caudate | 10 | 14 | 14 | 11 | 11.92 |

**Table S4.** CS+ > CS-, p < .001, uncorrected, k=5

**
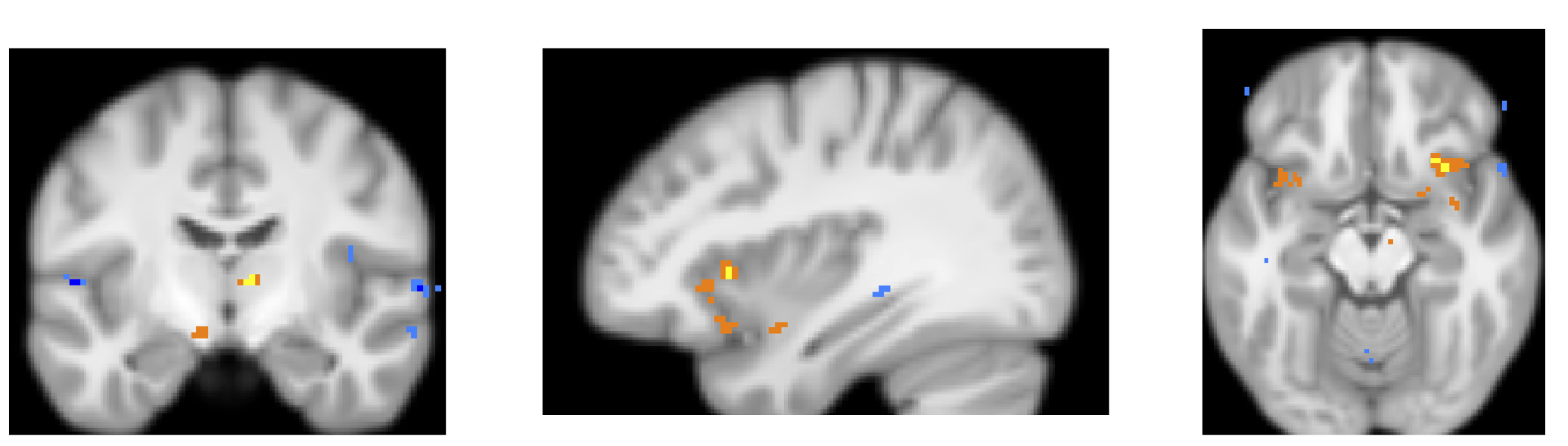
**

**Figure S3.** CS+ > CS-, p < .001, uncorrected (yellow), p < .005, uncorrected (orange), k > 5

| Region | X, Y, Z (MNI) | Cluster size (Voxels) | T-statistic |
| --- | --- | --- | --- |
| Insula | 30, 26, 0 | 11 | 8.31 |

**Table S5.** Whole-brain activity for CS+ >CS-, p <.05, FDR, k > 10

| Region | x | y | z | Cluster size (voxels) | T-statistic |
| --- | --- | --- | --- | --- | --- |
| **Reward Omission > Neutral Solution** | | |  | (voxels) |  |
| OFC/Frontal Operculum | 48 | 22 | -6 | 181 | 12.79 |
| Middle Temporal Gyrus | 60 | -44 | -2 | 81 | 11.72 |
| Middle Temporal Gyrus | 50 | -30 | -6 | 6 | 9.45 |
| Insular Cortex | -42 | 16 | -4 | 30 | 14.76 |
| Frontal Orbital Cortex | -44 | 22 | -8 | 7 | 10.26 |
| Frontal Pole | 44 | 54 | -2 | 17 | 12.25 |
| Frontal Pole | 34 | 52 | 4 | 6 | 10.95 |
| Inferior Frontal Gyrus | -54 | 22 | 10 | 23 | 13.17 |
| White Matter | 14 | -36 | 16 | 5 | 9.39 |
| White Matter/Thalamus | 18 | -12 | 18 | 6 | 8.95 |
| **Neutral Solution > Reward Omission** | | |  |  |  |
| Parahippocampal gyrus | -28 | -38 | -12 | 5 | 8.69 |
| Subcallosal cortex | 6 | 28 | -14 | 8 | 12.45 |
| Lateral Occipital Cortex | -52 | -62 | -6 | 30 | 10.28 |
| Paracingulate Gyrus | -10 | 36 | -6 | 78 | 13.29 |
| Cingulate gyrus | 2 | 38 | -4 | 9 | 8.69 |
| Paracingulate Gyrus | 8 | 46 | -6 | 5 | 11.12 |
| Posterior cingulate | 12 | -50 | 4 | 69 | 19.62 |
| Left Hippocampus | -20 | -40 | 4 | 16 | 10.84 |
| Cingulate gyrus/Precuneus | -8 | -52 | 4 | 37 | 11.47 |
| White Matter/Hippocampus | -20 | -44 | 4 | 5 | 9.54 |
| Paracingulate Gyrus | -2 | 56 | 4 | 8 | 8.5 |
| Occipital pole | 18 | -100 | 8 | 6 | 7.39 |
| Thalamus | -8 | -26 | 8 | 25 | 17.86 |
| Precuneus | -12 | -60 | 16 | 60 | 14.94 |
| Lateral Occipital Cortex | -42 | -68 | 12 | 40 | 18.05 |
| Lateral Occipital Cortex | -48 | -76 | 16 | 20 | 11.48 |
| Precuneus | 14 | -56 | 12 | 6 | 8.54 |
| Precuneus | 18 | -52 | 14 | 9 | 8.01 |

**Table S6.** Whole brain activity for Reward Omission > Neutral Solution, p < .001, uncorrected, k = 5

**
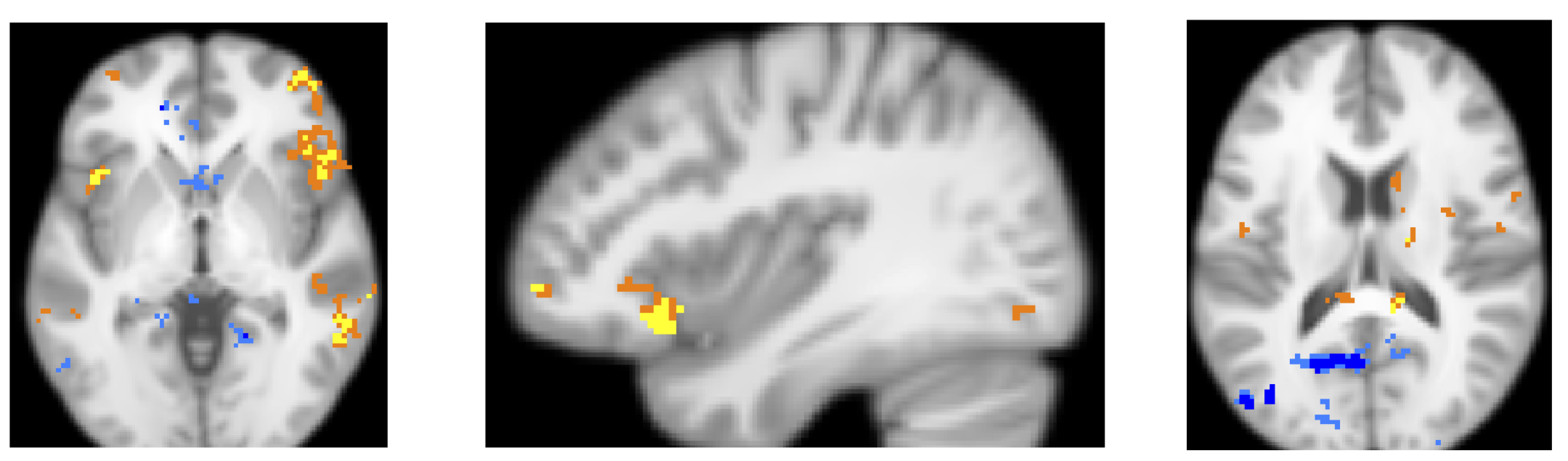
**

**Figure S4.** Reward Omission > Neutral Solution, p < .001, unc. (yellow), p < .005, unc. (orange), k > 5

| Region | x | y | z | Cluster size | T-statistic |
| --- | --- | --- | --- | --- | --- |
| **CS+ Inhibitor > Inhibitor** |  |  |  | (voxels) |  |
| Lingual gyrus | 2 | -82 | -4 | 157 | 7.72 |
| Fusiform gyrus | -26 | -74 | -10 | 7 | 5.83 |
| Intracalcarine cortex | -10 | -82 | 8 | 5 | 7.03 |

**Table S7.** Whole brain activity for CS+ and Inhibitor > Inhibitor, p < .05, FDR, k > 5

**Figure S5.**


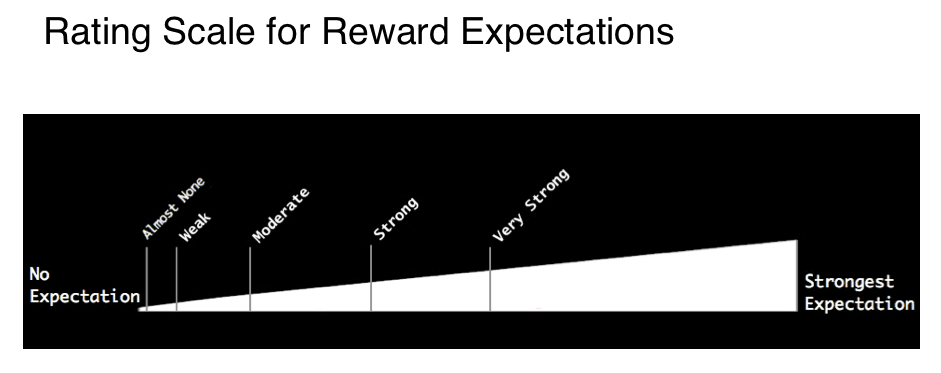


**Figure S6.** **Rating Scale for Reward Expectations.** Rating scale used for rating reward expectations. Each cue's level of reward expectation was rated from No Expectation to Strongest Expectation, with the scale markings following the Bartoshuk scale.

| All trials | CS  (A) | Control  (B) | CS+Inhib (AX) | Control (BY) | Inhibitor  (X) | Control 2  (Y) |
| --- | --- | --- | --- | --- | --- | --- |
| Block 1 | 24  (75% R, 25% NR) | 24  (100% NR) |  |  |  |  |
| Block 2 | 8  (75%R, 25% NR) | 8  (100% NR) | 8  (100% NR) | 8  (100% NR) |  |  |
| Blocks  3-5 | 12  (75% R,  25% NR) | 12  (100% NR) | 12  (100% NR) | 12  (100% NR) | 4  (100% NR) | 4  (100% NR) |
| Block 6 | 24  (75% R,  25% NR) | 24  (75% R,  25% NR) |  |  |  |  |

**Table S8.** Trial types in each block

**Figure S6:**

While the ROI analysis showed significant activity in the VTA ROI for the CS+ compared to the CS, this was only significant at an uncorrected threshold of p < .05, uncorrected for multiple comparisons. Below, we show activity in these regions at multiple thresholds, overlaid with both ROIs.


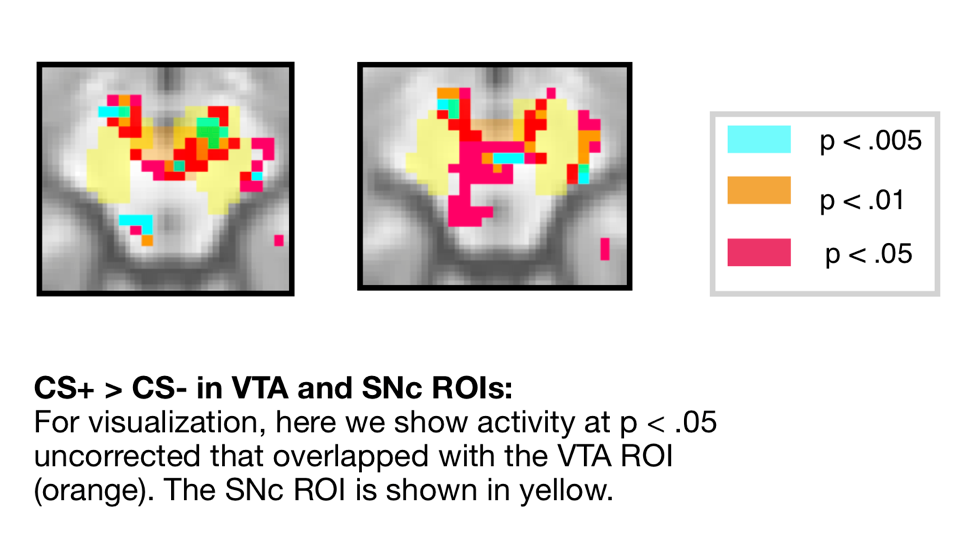

Supplement: Supplementary file 1 [file Table_1.docx]
